# Delusions of Vampirism in an Adolescent and Treatment With Clozapine: A Case Report

**DOI:** 10.7759/cureus.46352

**Published:** 2023-10-02

**Authors:** Catherine O'Brien, Brian Hallahan

**Affiliations:** 1 Child and Adolescent Psychiatry, Child and Adolescent Mental Health Services (CAMHS) Inpatient Unit, Health Service Executive (HSE) West, Galway, IRL; 2 Psychiatry, University of Galway, Galway, IRL

**Keywords:** capgras syndrome, childhood, clozapine, schizophrenia, vampirism

## Abstract

This case describes delusions of vampirism among several other psychotic symptoms in a 15-year-old who has a diagnosis of schizophrenia, with these delusions first presenting when he was 13 years of age. Delusions of vampirism can be associated with a strong desire to suck human blood but these delusional beliefs were not acted upon here. This is the first report of delusions of vampirism in childhood to date. The introduction of the antipsychotic medication clozapine after failed treatment trials with two other antipsychotic agents has been associated with a significant amelioration in symptomatology and an improvement in functioning.

## Introduction

Vampirism has been reported in the literature for centuries and is often affiliated with mythical horror figures [1], with ongoing interest in the vampire myth across popular culture [2]. Although vampirism is a pervasive legend, it can be associated with a severe mental disorder. Sometimes referred to as ‘Renfield’s syndrome’, it is characterized by a patient’s compulsion to consume blood, their own or another’s [3]. Delusions of vampirism are a rare phenomenon but have previously been reported in adults diagnosed with schizophrenia [4,5], with auto-vampirism (self-induced bleeding with a compulsion to consume one’s own blood) also noted [4]. Vampirism as a single entity (not related to psychosis) has also been suggested to be a sub-type of necrophilia [6]. Here, we present a case of delusions of vampirism, which have not been clearly reported to date in childhood and adolescence.

## Case presentation

We present the case of a 15-year-old boy, who has been an inpatient in a child and adolescent mental health unit for almost twelve months. He usually resides with his parents and older sibling. He first presented to the mental health service with psychotic symptoms in May 2022, after a referral from his general practitioner. His family had noticed a change in behavior for two to three months prior to his referral, which encompassed increased distress, poorer self-care, self-isolation, social withdrawal, reduced school attendance (he related to feeling physically unwell and consequently being unable to attend) with a deterioration noted in his academic performance in the 12-month period prior to his admission. Additionally, he was observed by his parents to be talking aloud despite no external stimuli, and on questioning by them, admitted to “hearing voices” but was reticent in discussing the details of same.

At initial and subsequent reviews early in his clinical history, he displayed multiple psychotic symptoms. He described delusions of vampirism, stating that he believed that he was a vampire but denied any strong thoughts or urges to suck people's blood. He believed being a vampire bestowed him with certain superpowers. These included an ability to “read people‘s minds” and engage in physical tasks, such as climbing buildings and high walls, akin to a superhero. He described the somatic delusion of an implant being present in his eye, which caused the iris of his eyes to change colors (blue to black) and allowed him to have superior vision. He stated this implant was further evidence of him being a vampire. He declined to wear glasses secondary to this belief, although he would usually require glasses for several daily tasks including watching television or engaging in school work.

He described many other polythematic delusions during the course of his inpatient admission. These include Capgras delusions of his family members being replaced by imposters (i.e. dead people), delusions of reference from characters on the television (i.e. usually news-readers and not fantasy-related characters), delusions of thought broadcasting, with many people knowing his inner thoughts and delusions of a persecutory nature (i.e. believing that his parents were against him and medications dispensed were poisonous and offered to him to cause him harm). Particularly, early in his illness course, he experienced multiple other delusions. These included nihilistic delusions (i.e. believing that part of his body was missing and that people near him were dead), somatic delusions (i.e. believed his legs were unequal in size, believed he was menstruating which he associated with abdominal pain and evidence that his gender was changing), delusions of love (i.e. staff members were in love with him), delusions of re-living events of the past, and a delusional mood. Formal thought disorder was evident with loosening of associations and tangentiality particularly prominent.

He described several perceptual abnormalities, including second and third-person auditory hallucinations of several people talking to him and about him, with running commentary also occasionally present. These auditory hallucinations were generally pleasant and not distressing, with him describing these perceptions as “his friends”. He hoped that antipsychotic medications would not reduce these and linked these perceptual abnormalities to other subjectively enjoyable experiences of having superpowers and “special missions”, with him frequently overtly responding to these hallucinations (i.e. laughing and talking aloud). Occasionally, these perceptions were distressing, including when “voices” made derogatory references to him being a vampire. Other perceptual abnormalities consisted of somatic hallucinations as described (i.e. internal body pains, the feeling of an implant in the eye). He denied gustatory or olfactory hallucinations.

The patient was not overtly distressed by these delusional beliefs and perceptions but did engage in some minor assaultive behaviors toward family members prior to admission, which he related to distress and not to beliefs of being a “vampire”. At times of heightened symptomatology, he often managed his distress via self-isolative measures by limiting contact with others and spending time alone in his room.

Observations from his appearance, behavior, and speech on review included him having a slouched posture, displaying minimal eye contact and engagement, apathy, paucity of speech, which was often monotonous, poor self-care, and reduced concentration evident.

After a brief initial period of treatment non-adherence, he was fully concordant with treatment. The antipsychotic medication risperidone was taken orally and increased over time to 6 mg per day. Due to the lack of any notable amelioration in any symptoms with this medication, he was prescribed olanzapine three months into his treatment with the dosage titrated up to 20 mg. There was a mild improvement in some symptoms, including a reduction in the intensity of his Capgras, Cotard’s, and somatic delusions (i.e., no longer described him menstruating or having a misshaped body); however, other symptoms, including delusions of vampirism were largely unchanged, with ongoing limited self-care and isolative behaviors evident. After three months of olanzapine administration, his treatment was changed to clozapine, with the dose of this medication titrated carefully as per Clozaril Patient Monitoring Service guidelines, to a total daily dose of 550 mg and a therapeutic level of 0.39 mg/L. Intensive nursing and occupational therapy support was provided to support self-care and provide a daily structure.

A very significant reduction in symptomatology has been noted following the initiation of clozapine, with a complete resolution of all delusional beliefs and a significant reduction in perceptual abnormalities with only occasional auditory hallucinations now present (i.e. running commentary). Increased social engagement has also been evident, with no formal thought disorder evident in either his speech or written notes. He currently engages in good self-care and is wearing glasses when required for certain tasks. He is due for discharge from hospital in the coming weeks (this has been occurring on a phased basis)

He has no previous history of alcohol or illicit psycho-active substance use and there is no family psychiatric history. Additionally, there were no cultural beliefs within his environment or family pertaining to vampires. As a very young child, he experienced approximately three febrile seizures that did not require treatment and have not recurred. He was diagnosed with a mild intellectual disability at 12 years of age with language levels consistent with cognitive level, and there was no history of a neurodevelopmental disorder (i.e. autism spectrum or attention deficit hyperactivity disorder).

Investigations

A full range of blood tests have been performed and are within the normal range (i.e. urea and electrolytes, liver function tests, glucose, prolactin, cholesterol, triglycerides, Troponin T, C-reactive protein) with full blood counts taken weekly due to his current treatment with clozapine, which again are with a normal range (i.e. white cell count, hemoglobin). An electrocardiogram (ECG) was conducted prior to medication commencement and has subsequently been repeated on a monthly basis with all recordings within the normal range. Physical examination for extra-pyramidal side effects and other metrics for metabolic syndrome (i.e. blood pressure and waist circumference) are undertaken on a regular basis with weight and median BMI monitored weekly (all within acceptable ranges and no significant changes evident to date). Magnetic resonance imaging (MRI) of the brain found no focal cerebral abnormality. An electroencephalogram (EEG) is awaited, but no seizure activity has been evident, despite close monitoring for the same. A positive and negative syndrome scale (PANSS) score [7], during the early phase of his admission was 146, with this currently reduced to 45.

Differential diagnosis

This gentleman has a diagnosis of schizophrenia according to the International Classification of Diseases 10 (ICD-10) and Diagnostic and Statistical Manual 5 (DSM-5). The schizoaffective disorder was considered a differential diagnosis due to some grandiose ideation pertaining to his delusional beliefs and some possible nihilistic delusions at times that individuals were no longer alive, however, no clear affective symptoms have been consistently evident.

Treatment

He is currently treated with clozapine 13550 mg per day as a monotherapy. Multi-disciplinary case conferences have been conducted on three occasions with senior clinical staff in attendance. Additionally, prior to the commencement of clozapine, a second opinion from a general adult psychiatrist (with significant experience in the management of psychosis was attained). Other supports include a mental health nurse, occupational therapy (inclusive of multiple social activities whilst in-patient), clinical psychology, social work support for his family, and dietetics input as required, with intensive dietary advice advised and a regular exercise plan provided. Schooling has been provided throughout his admission. On discharge from the hospital, he will attain regular outpatient medical reviews, community mental health nursing input, clozapine nursing staff support (including fortnightly blood tests to examine his white cell count), and social work input for his family. Regular metabolic monitoring (lipid, glucose, and blood pressure) will additionally be conducted.

Outcome and follow-up

The current treatment regime is proving successful after failed treatment attempts with two other antipsychotic agents, both administered at an optimal dose for over six weeks. He will require significant input in the community and his prognosis is guarded due to the longevity of his symptoms and early age of symptom onset. However, his symptoms have largely abated and ongoing support (particularly at times of distress) will help support this patient’s recovery.

Cognitive behavior therapy for psychosis would be optimal for this patient and will be provided in the coming months with the aim that this intervention will further ameliorate his psychotic symptoms and increase his insight, which currently remains quite limited. Supported school placement and engagement with his family (i.e. family and behavior therapy) will also be provided and will be important to reduce his risk of relapse.

## Discussion

In this case report, we present a patient who had multiple psychotic symptoms, including prominent delusions of vampirism with an early age of onset and symptoms first presenting at 13 years of age. This patient was diagnosed with treatment-resistant schizophrenia at 14 years of age. To our knowledge, this is the first case of delusions of vampirism in a child. Kayton, in his detailed description of vampirism and its association with mental health disorders, described a case of a 15-year-old who had a preoccupation with mirrors (mirror sign), with the author stating that this sign related to “a retreat into fantasy would make her fearful of stepping through the looking glass”; however delusions of vampirism were not clearly described in this case [8].

Delusions of vampirism (transforming into a vampire) are rare and can be conceptualized as a delusion of misidentification of self. This patient also misidentified others early in his illness course (i.e. Capgras syndrome). The individual described in this case report has not acted on these delusions, despite the additional presence of multiple other psychotic symptoms, including auditory hallucinations stating that he is a vampire. Of note, he has not described experiencing command auditory hallucinations instructing him to consume human blood. This lack of engagement in acting on these beliefs is consistent with a recent case [9], where a patient with delusions of vampirism displayed no aggressive behaviors secondary to these beliefs. This is in contrast to a reported case of auto-vampirism, where the patient consumed their own blood [4], or reports of vampirism where the blood of others was consumed [8].

The causation of delusional misidentification of self remains not fully understood. Where these delusions occur in individuals with focal neurological lesions, studies have noted a preponderance of right frontal and parietal lobe lesions, but acknowledge a greater distribution of lesions may be important in delusion formation [10-12]. One theory surmises that an impairment in processing sensory input combined with an impairment in our belief evaluation system, located in the right frontal lobe, can result in the persistence of such delusional beliefs [13]. Additionally, culture has been long understood to play an integral role in the content of delusions, with recent years noting a significant increase in the vampire myth being a component of popular culture, likely related to the “Twilight Saga” series of books and an array of films [2].

Delusions of vampirism are not pathognomonic and can be viewed as a symptom(s) of an underlying neurological or psychiatric condition. Delusions of vampirism have most commonly been related to schizophrenia or schizophreniform disorders [4,5,9] but have been noted to occur as a single entity (not related to psychosis [8] and have been suggested to be a sub-type of necrophilia when this is the case [6].

Clozapine is utilized for the management of treatment-resistant schizophrenia but is rarely utilized in children and adolescents, particularly as it is only licensed for use in patients aged 16 years and over [14], rendering its use in younger children unlicensed. Other potential reasons for clozapine’s limited utilization may relate to the low incidence of schizophrenia in younger adolescents, serious adverse effects such as neutropenia (albeit rare), and the requirement for frequent venepuncture (at least weekly for 18 weeks) [15]. Additionally, current evidence suggests that children are potentially more likely to be more sensitive to the neutropenic effects of clozapine, particularly if they are of male gender [16]. Other frequent adverse effects such as hypersalivation and weight gain, may additionally be more problematic in adolescents and young adults [17]. Despite these concerns, a number of studies including two small randomized controlled trials in childhood-onset schizophrenia have noted greater therapeutic benefits with clozapine compared to other antipsychotic agents [18,19], with evidence that clozapine can be a uniquely beneficial second-line antipsychotic agent for treating children or adolescents with refractory schizophrenia [20]. The dose of clozapine chosen for this study was based on clinical response, tolerability, and plasma levels, which were within the normal range. Of note, this dose was higher than the mean doses utilized in some previous studies evaluating clozapine in adolescents. For example, the mean dose of clozapine was 327 mg in a study by Shaw et al. (2006) although their patient cohort was on average 11.7 years younger than the patient presented in this case report [18].

## Conclusions

This case suggests that delusions of vampirism can occur during childhood, in the context of schizophrenia. Clozapine administration was associated with a significant amelioration in symptomatology and should be considered in the absence of response from other antipsychotic medications, even in children and adolescents under the age of 16 years.

## References

[1] Hemphill RE, Zabow T (1983). Clinical vampirism. A presentation of 3 cases and a re-evaluation of Haigh, the 'acid-bath murderer'. SA Med J.

[2] Rainford AC, Flaherty GT, Hallahan B (2023). Vampire tourism and vampirism: the darker side of travel medicine. J Travel Med.

[3] Mac Suibhne S, Kelly D (2010). Vampirism as mental illness: myth, madness and the loss of meaning in psychiatry. Soc History Med.

[4] Jensen HM, Poulsen HD (2002). Auto-vampirism in schizophrenia. Nord J Psychiatry.

[5] Benezech M, Bourgeois M, Boukhabza C (1981). Cannibalism and vampirism in paranoid schizophrenia. J Clin Psychiatry.

[6] Rosman J, Resnick P (1989). Sexual attraction to corpses: a psychiatric review of necrophilia. Bull Am Acad Psychiatr Law.

[7] Kay SR, Fiszbein A, Opler LA (1987). The positive and negative syndrome scale (PANSS) for schizophrenia. Schizophr Bull.

[8] Kayton L (1972). The relationship of the vampire legend to schizophrenia. J Youth Adolesc.

[9] Mulholland K, McLoughlin A, McDonald C, Hallahan B (2023). Kynanthropic and vampirism delusions: a case report and review of the literature. Ir J Psychol Med.

[10] Devinsky O (2009). Delusional misidentifications and duplications: right brain lesions, left brain delusions. Neurology.

[11] Thode KI, Faber RA, Chaudhuri TK (2012). Delusional misidentification syndrome: right-hemisphere findings on SPECT. J Neuropsychiatry Clin Neurosci.

[12] Darby R, Prasad S (2016). Lesion-related delusional misidentification syndromes: a comprehensive review of reported cases. J Neuropsychiatry Clin Neurosci.

[13] Coltheart M, Langdon R, McKay R (2007). Schizophrenia and monothematic delusions. Schizophr Bull.

[14] Clozaril 25 mg tablets. https://www.medicines.org.uk/emc/product/4411/smpc#gref.

[15] Gilboy S, Hollywood E (2009). Helping to alleviate pain for children having venepuncture. Paediatr Nursing.

[16] Maher KN, Tan M, Tossell JW (2013). Risk factors for neutropenia in clozapine-treated children and adolescents with childhood-onset schizophrenia. J Child Adolesc Psychopharmacol.

[17] Remschmidt H, Fleischhaker C, Hennighausen K, Schulz E (2000). Management of schizophrenia in children and adolescents. The role of clozapine. Paediatr Drugs.

[18] Shaw P, Sporn A, Gogtay N (2006). Childhood-onset schizophrenia: a double-blind, randomized clozapine-olanzapine comparison. Arch Gen Psychiatry.

[19] Frazier JA, Cohen LG, Jacobsen L (2003). Clozapine pharmacokinetics in children and adolescents with childhood-onset schizophrenia. J Clin Psychopharmacol.

[20] Gogtay N, Rapoport J (2008). Clozapine use in children and adolescents. Expert Opin Pharmacother.

